# Efficacy and safety of PARP inhibitors in older patients with advanced ovarian cancer: a systematic review and network meta-analysis

**DOI:** 10.3389/fonc.2026.1875768

**Published:** 2026-07-09

**Authors:** Lei Liang, Bo Yang, Yuanyuan Wu, Jing-Lei Liu, Rongna Liu, Li Sun

**Affiliations:** Department of Obstetrics and Gynaecology, 980 (Bethune International Peace) Hospital of PLA Joint Logistics Support Forces, Shijiazhuang, Hebei, China

**Keywords:** advanced ovarian cancer, chemotherapy, gynaecologic malignancy, network meta-analysis, PARP inhibitors

## Abstract

**Background:**

Older adults with advanced ovarian cancer (AOC) are underrepresented in randomized clinical trials (RCTs), limiting age-specific evidence on poly(ADP-ribose) polymerase inhibitors (PARPi). This network meta-analysis (NMA) compares PARPi efficacy and safety across age groups.

**Methods:**

We searched PubMed, Embase, and Web of Science until January 20, 2026, for RCTs evaluating PARPi in adults with AOC. Screening and extraction utilized Nested Knowledge. Risk of bias was assessed via RoB 2. A frequentist NMA estimated hazard ratios (HR) and odds ratios (OR) with 95% CIs. Treatment rankings utilized P-scores.

**Results:**

A total of 13 RCTs were included. In younger patients, olaparib (HR, 0.32; 95% CI, 0.19–0.54), rucaparib (HR, 0.33; 95% CI, 0.16–0.69), and niraparib (HR, 0.53; 95% CI, 0.38–0.74) significantly improved progression-free survival (PFS) compared with placebo or chemotherapy. Similar benefits were observed in older patients (≥65 years), with significant PFS improvements for olaparib (HR, 0.44; 95% CI, 0.25–0.77), rucaparib (HR, 0.43; 95% CI, 0.19–0.97), and niraparib (HR, 0.56; 95% CI, 0.38–0.83). In the overall adult population, senaparib, veliparib, and olaparib were associated with significant improvements in PFS. Regarding safety, niraparib was associated with increased odds of treatment-emergent adverse events (OR, 3.80; 95% CI, 1.72–8.39), while both niraparib and olaparib were associated with higher risks of grade ≥3 anaemia and other hematologic toxicities. Placebo or chemotherapy ranked most favourably across most safety outcomes. Substantial heterogeneity was observed across several efficacy networks, and most treatment comparisons relied on indirect evidence.

**Conclusions:**

PARP inhibitors improved progression-free survival across age groups, including patients aged ≥65 years. However, treatment was associated with increased hematologic and gastrointestinal toxicities. Further studies should assess geriatric outcomes, long-term safety, and patient-reported outcomes.

## Introduction

Ovarian cancer is a major contributor to cancer-related deaths among women, primarily due to the fact that the majority of cases are identified only after the disease has progressed to an advanced stage (1). Nonspecific symptoms, absence of effective screening strategies, and aggressive tumour biology contribute to delayed diagnosis and poor prognosis (2, 3). Age is a key risk factor for ovarian cancer, with incidence and mortality increasing steadily with advancing age (4). A large proportion of patients are diagnosed after the age of 60 years, and many present with advanced disease requiring systemic therapy (5, 6). As global populations continue to age, the number of older patients with advanced ovarian cancer is expected to rise, emphasizing the need for evidence that adequately reflects outcomes across different age groups (7). Despite this demographic reality, older patients remain underrepresented in randomized clinical trials (RCTs), resulting in limited age-specific data and uncertainty regarding the generalizability of trial findings to routine clinical practice (8, 9).

Poly (ADP-ribose) polymerase (PARP) inhibitors have substantially transformed the treatment landscape of advanced ovarian cancer (AOC), especially when used as maintenance therapy after patients respond to platinum-based chemotherapy (10, 11). By targeting defects in DNA damage repair pathways, these agents have demonstrated consistent improvements in progression-free survival (PFS) (12, 13). Their clinical benefit has led to widespread adoption in both newly diagnosed and recurrent disease settings (14). However, most pivotal trials enrolled relatively fit patients, often excluding individuals with significant comorbidities or impaired functional status, characteristics more commonly observed in older adults (15, 16). Age-related physiologic changes, including altered drug metabolism and reduced bone marrow reserve, may affect both treatment efficacy and tolerability (17, 18). In addition, older patients are more likely to experience polypharmacy and comorbid conditions, which may increase susceptibility to adverse events and treatment discontinuation (19). These factors raise important concerns regarding whether the benefits observed in younger populations are maintained in older patients and whether toxicity profiles differ meaningfully by age (20, 21).

Safety considerations are particularly relevant when treating older patients with PARP inhibitors (22, 23). Hematologic adverse events such as anaemia, thrombocytopenia, and neutropenia, along with nonhematologic toxic effects including fatigue and gastrointestinal symptoms, may have a greater clinical impact in older adults (24, 25). Even moderate toxic effects can compromise functional independence, adherence, and quality of life (QoL) in this population (26, 27). Although subgroup analyses by age have been reported in some trials, these analyses are often exploratory, inconsistently defined, and insufficiently powered to draw firm conclusions (28, 29). Moreover, direct comparative data among different PARP inhibitors in older patients are lacking (22, 30). To address these limitations, this network meta-analysis (NMA) pooled the data from randomized clinical trials (RCTs) by age group, including younger and older adult patients aged 18 years and above. By integrating direct and indirect evidence, NMA enables comparative evaluation of multiple PARP inhibitors in the absence of head-to-head trials. This age-stratified approach aims to clarify differences in efficacy and safety across age categories and to provide evidence that supports informed, individualised treatment decisions for older patients with advanced ovarian cancer.

## Methods

This NMA was conducted according to the PRISMA extension for network meta-analyses (**Supplementary Table 1**). Nested Knowledge software was used for study screening and data management.

### Eligibility criteria

We included randomised trials enrolling adults aged ≥18 years, younger and older (≥65 years) with AOC, including epithelial ovarian, fallopian tube, or primary peritoneal cancer. Studies were eligible if outcomes could be analysed by age group. PARP inhibitors were used, administered alone or with standard therapy, and compared with placebo, observation, chemotherapy, or another PARP inhibitor. Eligible studies reported at least one efficacy or safety outcome and provided sufficient data for analysis. Trials limited to early-stage disease or nonadult populations were excluded, as were observational studies, reviews, animal studies, and case reports (**Supplementary Table 2**).

### Search strategy

We searched PubMed, Embase, and Web of Science from database inception through January 20, 2026. Keywords related to ovarian cancer (ovarian neoplasms, ovarian cancer, and ovarian carcinoma) were combined with terms for PARP inhibitors, including niraparib, olaparib, rucaparib, and talazoparib. Boolean operators (OR and AND) were used to combine disease- and intervention-related terms, and we also manually screened reference lists and relevant systematic reviews and meta-analyses focusing on advanced ovarian cancer (**Supplementary Table 3**).

Age-specific subgroup data were extracted during study screening and data extraction rather than through database-level age filtering.

### Screening and data extraction

We imported all records into the Nested Knowledge software for screening and data management. Duplicate literature was removed automatically by the software. Two reviewers screened records based on predefined eligibility criteria during the first-pass screening. In the second-pass screening, full texts were reviewed by 2 reviewers. A third reviewer resolved any disagreements.

Data extraction was carried out independently by two reviewers using a standardised data collection form. The extracted information included study design, country of origin, and patient age, follow-up duration, and intervention and comparator details, as well as efficacy and safety outcomes (progression-free survival, grade ≥3 anaemia, any treatment-emergent adverse events, anaemia, neutropenia, thrombocytopenia, nausea, vomiting, constipation, dose reduction, dose interruption, and treatment discontinuation). Discrepancies were resolved through discussion by a third reviewer.

### Quality assessment

The methodological quality of the included studies was evaluated using the Cochrane Risk of Bias 2 (RoB 2) tool (31). Each trial was assessed using domain-specific signalling questions covering the randomisation, deviations from intended interventions, missing outcome data, measurement of outcomes, and selective reporting of results. Two reviewers independently assessed each domain and assigned an overall risk-of-bias judgment: low risk, some concerns, or high risk. Disagreements were resolved through discussion, and when necessary, by consulting a third reviewer.

### Statistical analysis

All analyses were performed within a frequentist framework using R software (32). Data were pooled to estimate Hazard ratios (HR) or odds ratios (OR) with corresponding 95% CIs for efficacy and safety outcomes. Between-study heterogeneity was assessed using the I² statistic and the Cochran Q test (33). Formal inconsistency testing was limited because most treatment networks exhibited star-shaped geometries without closed loops, precluding robust node-splitting or loop-specific inconsistency assessment. A connected treatment network was constructed, and comparative effects were summarised using league tables. Treatment rankings were based on P-scores, with values closer to 1 indicating a higher-ranked treatment and values closer to 0 indicating a lower-ranked treatment (34). Age-based subgroup analyses were conducted. Influence analysis was performed for outcomes including more than five studies using the netimpact() function in the netmeta package. Meta-regression analyses were not performed because of the limited number of studies available within each subgroup and the sparse network geometry. To maintain a connected treatment network, placebo- and chemotherapy-containing control regimens were grouped within a common comparator node. This approach was adopted to preserve network connectivity and facilitate indirect comparisons among PARP inhibitors.

## Results

**Figure 1:**
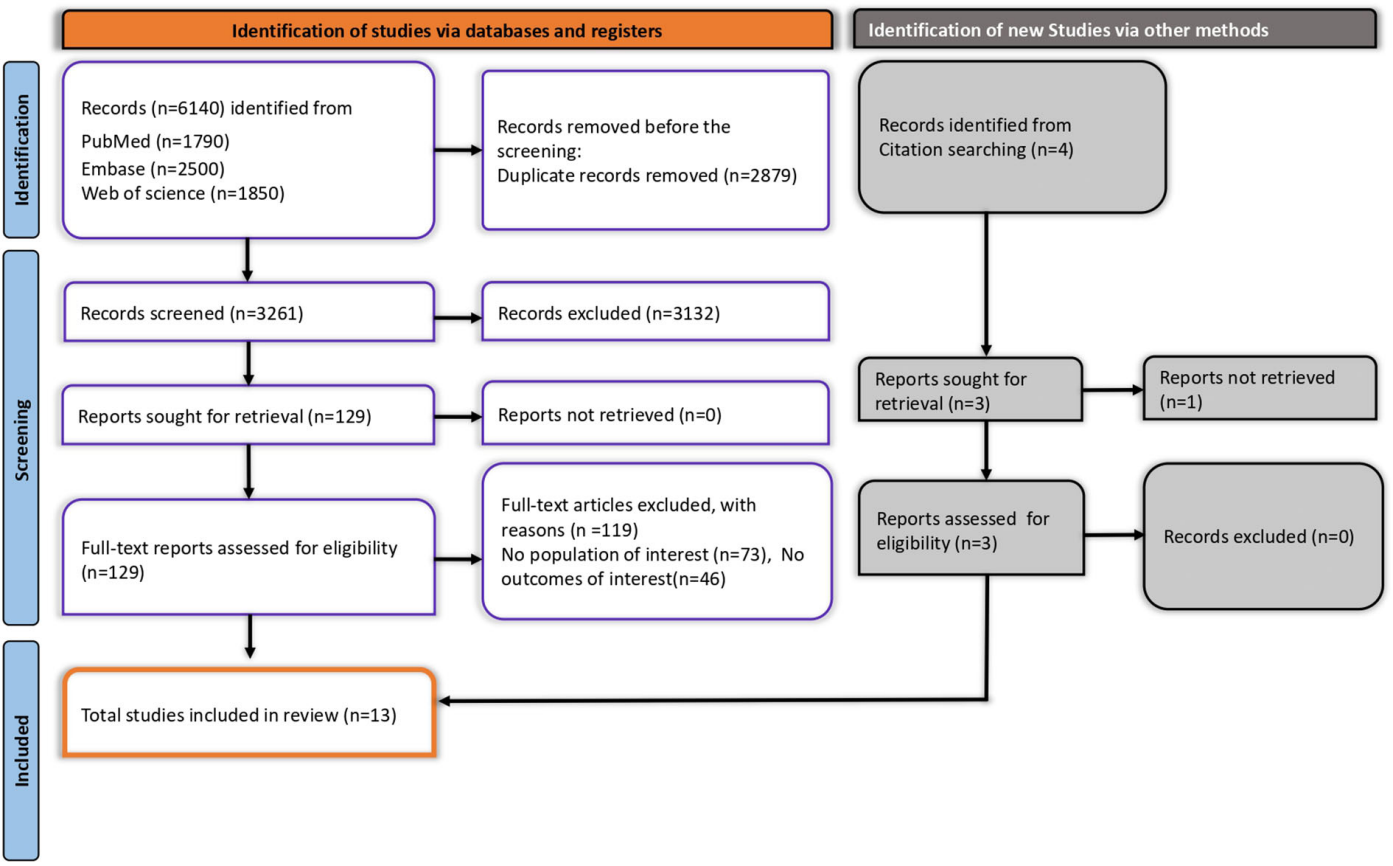
PRISMA flow diagram depicting study selection.

Of the 6140 records identified, 2879 duplicates were removed, leaving 3261 for screening. After excluding 3132 records, 129 full-text articles were assessed. Of these, 119 were excluded due to irrelevant population (73) or outcomes (46). Citation searching found 4 additional records; 3 were assessed, and 1 was not retrieved. In total, 13 studies were included (**Figure 1**).

### Characteristics of included studies

We included phase III RCTs conducted in multiple countries, including the United States(USA) (35, 36), Germany (37), Japan (38, 39), Italy (40), Spain (41), Denmark (42), China (43, 44), Sri Lanka (45), and London (37). Most trials enrolled adult patients with AOC, with several studies reporting outcomes stratified by age (<65 years vs≥65 years), while others enrolled adults aged 18 years or older. Follow-up duration varied across studies, ranging from approximately 22 months to more than 5 years.

**Figure 2:**
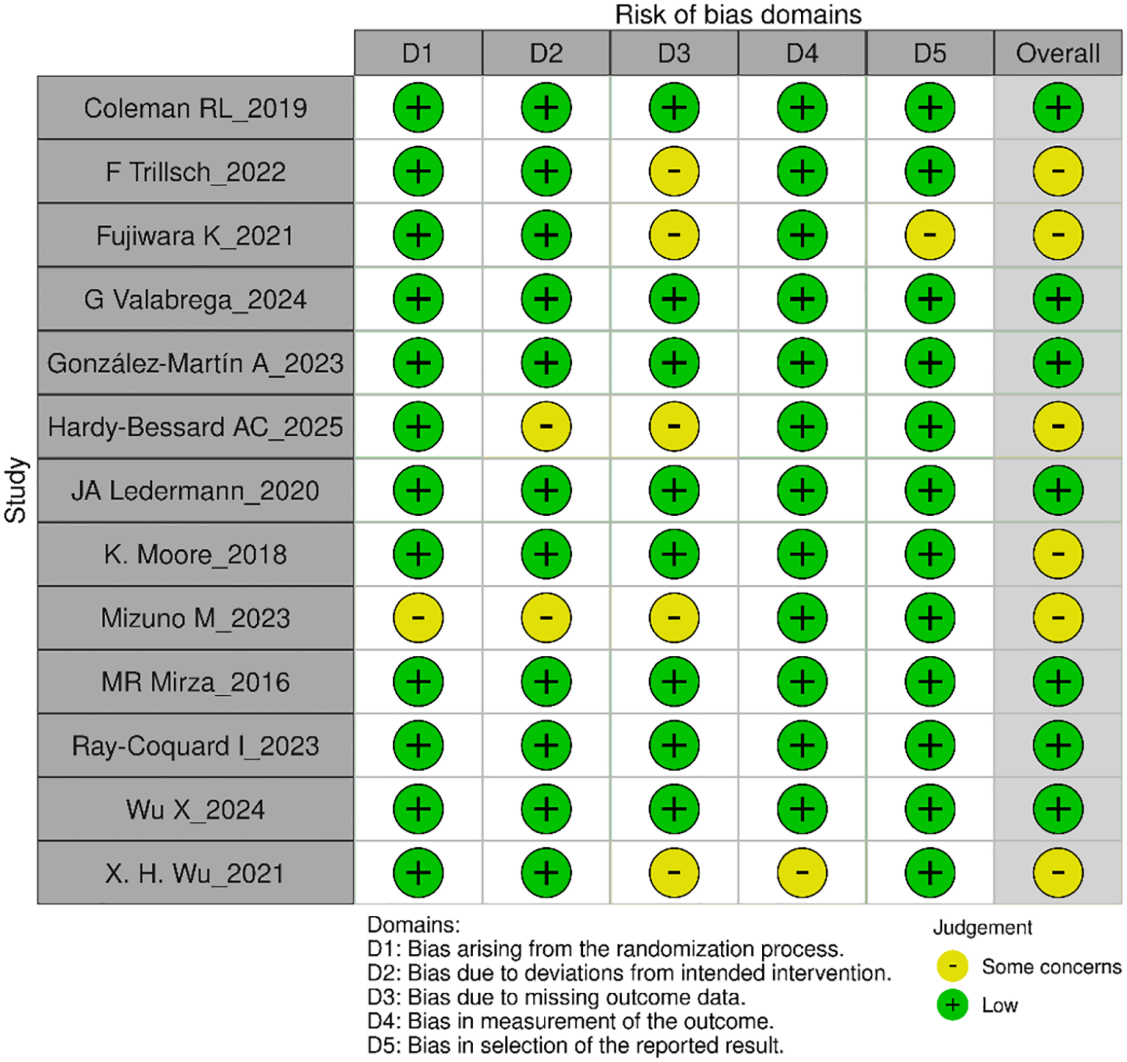
Risk of bias across included trials using RoB 2.

Sample sizes differed substantially across trials, with intervention groups ranging from approximately 60 to more than 500 patients. Evaluated interventions included olaparib, niraparib, rucaparib, veliparib, and senaparib, administered as maintenance therapy or as part of combination regimens. Comparator groups most commonly received placebo, placebo plus bevacizumab, or chemotherapy-based regimens (**Table 1**). The risk of bias was generally low, with only minor concerns related to missing outcome data or deviations from planned interventions, and no study was judged to have high overall bias (**Figure 2**).

**Table 1:** Characteristics of included studies.

Author (year)	Study design	Country	Age (years)	Follow-up(months)	Number of participants in the intervention and control groups	Intervention details	Comparator details
Coleman RL_2019 (35)	Phase III RCT	USA	<65 years ≥65 years	–	Intervention 382; Control 383	Veliparib-combination → veliparib maintenance	Veliparib + Chemo
F Trillsch_2022 (46)	Phase III RCT	Germany	–	–	Intervention -; Control -	Olaparib	Placebo
Fujiwara K_2021 (38)	Phase III RCT	Japan	<65 years ≥65 years	27 months	Intervention 15; Control 9	Maintenance olaparib tablets 300 mg twice daily	placebo twice daily, plus bevacizumab
G Valabrega_2024 (40)	Phase III RCT	Italy	<65 years ≥65 years	40–48 months	Intervention 487; Control 246	Niraparib	Placebo
González-Martín A_2023 (41)	Phase III RCT	Spain	≥18 years	41 months	Intervention 487; Control 246	Niraparib	Placebo
Hardy-Bessard AC_2025 (36)	Phase III RCT	USA	<65 years ≥65 years		Intervention 200; Control 200	Placebo with niraparib maintenance	Dedostarlimab with dostarlimab niraparib -maintenance
JA Ledermann_2020 (37)	Phase III RCT	London	<65 years ≥65 years	28 months	Intervention 375; Control 189	Rucaparib	Placebo
K. Moore_2018 (45)	Phase III RCT	Sri Lanka	<65 years ≥65 years	41 months	Intervention 260; Control 131	Olaparib 600 mg/daily	Placebo
Mizuno M_2023 (39)	Phase III RCT	Japan	<65 years ≥65 years	–	Intervention ~60; Control ~50	Veliparib-throughout	veliparib-combination
MR Mirza_2016 (42)	Phase III RCT	Denmark	≥18 years	22 months	Intervention 372; Control 181	Niraparib	Placebo
Ray-Coquard I_2023 (47)	Phase III RCT	Germany	≥18 years	≥ 5 years	Intervention 537; Control 269	Olaparib (300 mg twice daily plus bevacizumab (15 mg/kg every 3 weeks; 15 months total)	Placebo + bevacizumab
Wu X_2024 (43)	Phase III RCT	China	≥18 years		Intervention 271; Control 133	Senaparib	Placebo
X. H. Wu_2021 (44)	Phase III RCT	China	≥18 years	< 6–12 OR ≥ 12 months)	Intervention 177: Control 88	Niraparib	Placebo

### Network meta-analysis

#### Structure of the NMA

**Figure 3:**
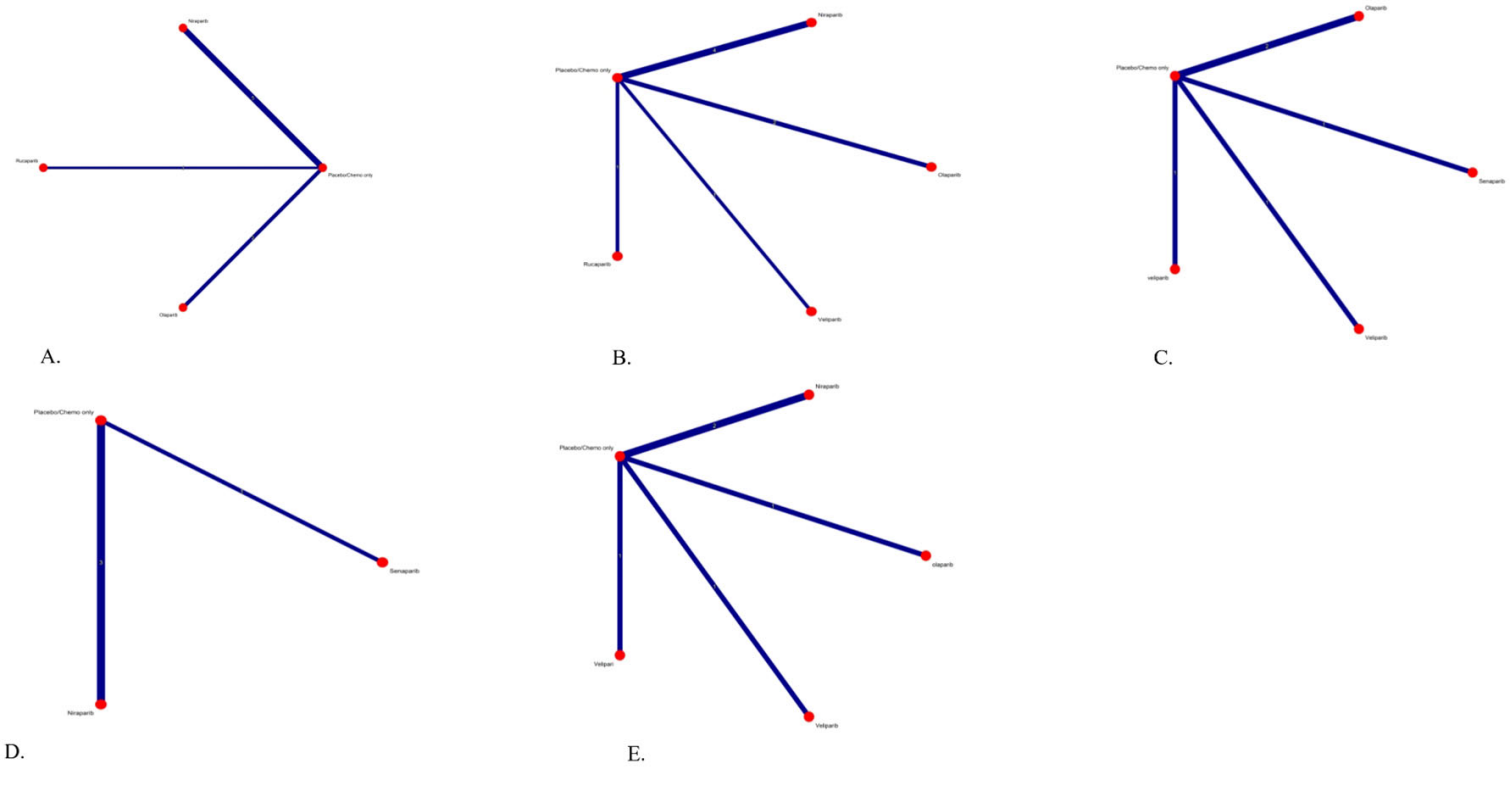
Primary end points network plots. **(A)** Young age PFS, **(B)** Old age PFS, **(C)** ≥18 years PFS, **(D)** Any treatment-emergent adverse events (TEAEs), **(E)** Grade ≥3 anemia.

**Figure 3** presents the network plots for progression-free survival in younger patients, older patients, and adults aged ≥18 years, as well as grade ≥3 anaemia. Additional network plots were generated for hematologic toxicities (neutropenia and thrombocytopenia), gastrointestinal toxicities (nausea, vomiting, constipation, diarrhoea, and abdominal pain), treatment discontinuation, and dose reduction due to treatment-emergent adverse events (**Supplementary Figure 3**). Across all analyses, placebo or chemotherapy was the common comparator. No direct head-to-head comparisons between PARP inhibitors were identified. Most networks exhibited a predominantly star-shaped geometry, with limited direct evidence among active treatments.

#### Progression-free survival

**Figure 4:**
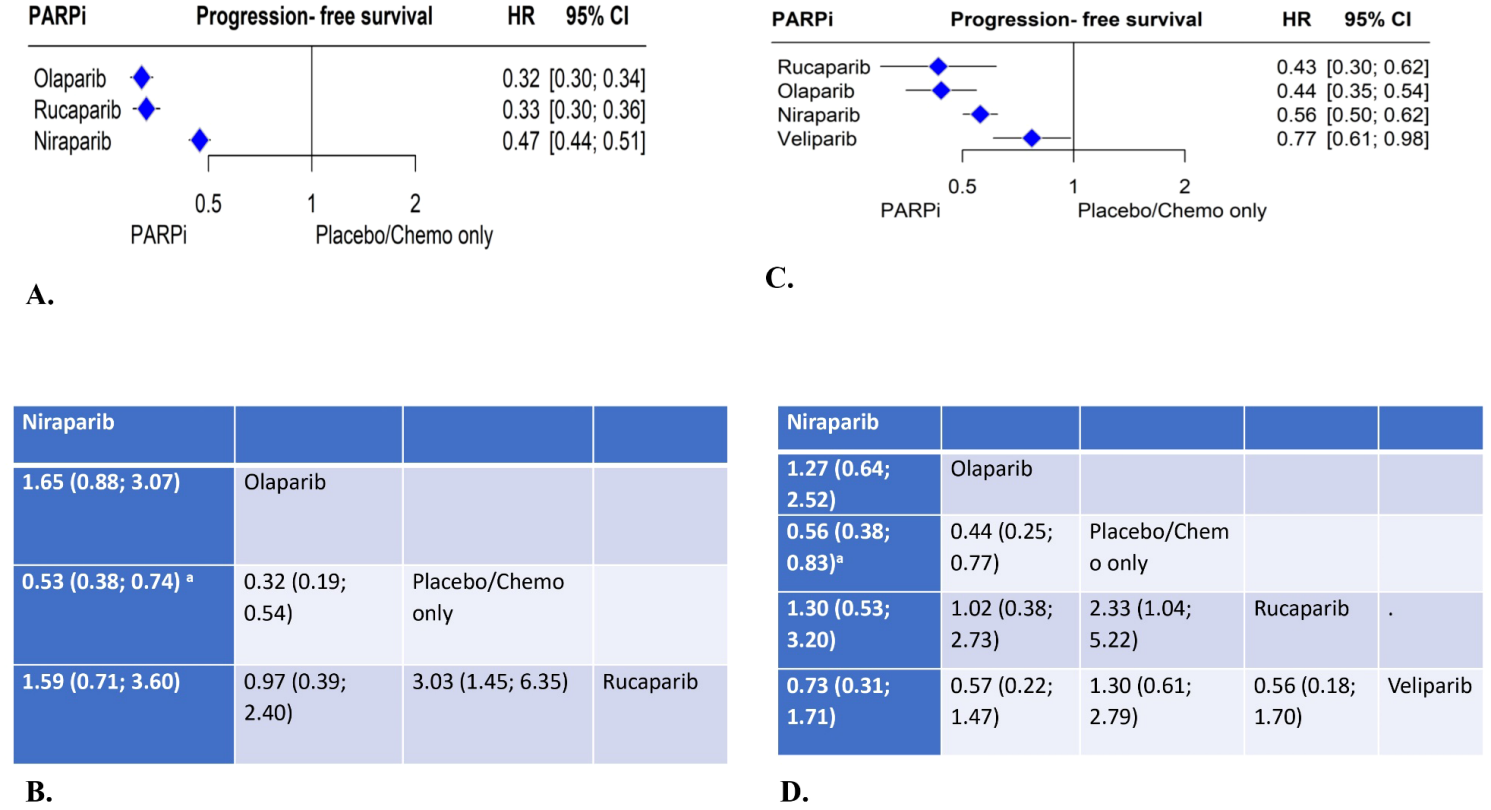
Primary end points: forest plot and league tables. **(A)** Progression-free analysis (Young age): PPRPi v/s Placebo/Chemo only, **(B)** Progression-free analysis (Old age): PPRPi v/s Placebo/Chemo only, **(C)** Progression-free analysis (Young age): PPRPi Strategy, **(D)** Progression-free analysis (Old age): PPRPi Strategy.

We pooled pairwise comparisons in younger patients (8 RCTs; 4 treatments), showed significant improvements in PFS for olaparib compared with placebo or chemotherapy (HR, 0.32; 95% CI, 0.19–0.54), rucaparib compared with placebo or chemotherapy (HR, 0.33; 95% CI, 0.16–0.69), and niraparib compared with placebo or chemotherapy (HR, 0.53; 95% CI, 0.38–0.74). Olaparib was also favoured over niraparib (HR, 0.53; 95% CI, 0.38–0.74). All remaining pooled pairwise comparisons were not statistically significant (**Figures 4A, B**). Heterogeneity was high (I² = 96.2%; P <.001), with no heterogeneity between designs (P = 0).

In older patients (8 RCTs; 5 treatments), statistically significant pooled comparisons favoured olaparib compared with placebo or chemotherapy (HR, 0.44; 95% CI, 0.25–0.77), rucaparib compared with placebo or chemotherapy (HR, 0.43; 95% CI, 0.19–0.97), and niraparib compared with placebo or chemotherapy (HR, 0.56; 95% CI, 0.38–0.83). Indirect comparisons also favoured olaparib and niraparib over placebo or chemotherapy. All other pooled indirect comparisons were not statistically significant (**Figure 4C, D**). Heterogeneity was substantial (I² = 90.1%; P <.001), with no heterogeneity between designs (P = 0).

Among adults aged ≥18 years (18 year or older) (5 RCTs; 4 treatments), statistically significant pooled comparisons favoured senaparib compared with placebo or chemotherapy (HR, 0.43; 95% CI, 0.38–0.49), veliparib compared with placebo or chemotherapy (HR, 0.46; 95% CI, 0.28–0.75), and olaparib compared with placebo or chemotherapy (HR, 0.49; 95% CI, 0.30–0.80). Indirect comparisons were consistent with these findings, while all other pairwise comparisons were not statistically significant. Heterogeneity was substantial (I² = 77.1%; P = .01), with no heterogeneity between designs (P = 0).

### Safety outcomes for adults aged ≥18 years (18 or older)

#### Any treatment-emergent adverse events

For any TEAEs (4 RCTs; 3 treatments; 2558 patients), pooled comparisons showed higher odds with niraparib compared with placebo or chemotherapy (OR, 3.80; 95% CI, 1.72–8.39). Comparisons involving senaparib were not significant, and no significant differences were observed between active treatments (**Supplementary Figure 1**).

#### Grade 3 or higher anaemia

For grade 3 or higher anaemia (5 RCTs; 5 treatments; 2649 patients), pooled comparisons showed higher odds with olaparib compared with placebo or chemotherapy (OR, 17.57; 95% CI, 4.21–73.24) and with niraparib compared with placebo or chemotherapy (OR, 23.61; 95% CI, 7.54–73.94). Veliparib was also associated with higher odds compared with placebo or chemotherapy, with smaller effect estimates. Comparisons between PARP inhibitors favoured the control group (**Supplementary Figure 2**). Heterogeneity was substantial (I² = 77.1%; P = .01), with no heterogeneity between designs (P = 0).

### Hematologic toxicity

Any-grade neutropenia (5 RCTs; 5 treatments; 2787 patients) was significantly more frequent with niraparib (OR, 6.62; 95% CI, 3.46–12.68), olaparib (OR, 2.30; 95% CI, 1.25–4.24), senaparib (OR, 6.97; 95% CI, 4.40–11.06), and veliparib (OR, 1.44; 95% CI, 1.15–1.81) versus placebo or chemotherapy. In indirect comparisons, niraparib was associated with higher odds than olaparib (OR 2.88; 95% CI 1.18–7.02) and veliparib (OR 4.58; 95% CI 2.30–9.12). For grade ≥3 neutropenia (5 RCTs; 5 treatments; 2635 patients), pooled comparisons showed higher odds with niraparib versus placebo or chemotherapy (OR, 6.62; 95% CI, 3.46–12.68), whereas veliparib showed higher odds (OR, 16.29; 95% CI, 8.16–32.53). Indirect analyses further demonstrated higher odds with niraparib compared with olaparib (OR 3.47, 95% CI 1.12–10.76) and senaparib (OR 9.23, 95% CI 3.83–22.27). Veliparib exhibited the most favourable profile, with lower odds than placebo or chemotherapy (OR 0.06, 95% CI 0.03–0.12) (**Supplementary Figure 4**).

Any-grade thrombocytopenia was evaluated across 5 RCTs (2,781 patients). Compared with placebo or chemotherapy, significantly increased risks were observed with niraparib (OR 26.78, 95% CI 13.68–52.41), olaparib (OR 3.14, 95% CI 1.19–8.31), senaparib (OR 16.16, 95% CI 9.40–27.78), and veliparib (OR 3.04, 95% CI 2.45–3.77). Niraparib was associated with higher odds than both olaparib (OR 8.53, 95% CI 2.61–27.85) and veliparib (OR 8.81, 95% CI 4.35–17.83). Olaparib demonstrated a lower risk than senaparib (OR 0.19, 95% CI 0.06–0.59). For grade ≥3 thrombocytopenia (4 RCTs; 2,384 patients), marked increases in risk were observed with niraparib (OR 90.83, 95% CI 12.57–656.11) and veliparib (OR 5.63, 95% CI 4.13–7.67) relative to placebo or chemotherapy. Indirect comparisons showed substantially higher odds with niraparib compared with olaparib (OR 183.08, 95% CI 11.22–2987.22) and veliparib (OR 16.14, 95% CI 2.18–119.43), while olaparib demonstrated lower odds than veliparib (OR 0.09, 95% CI 0.01–0.65) (**Supplementary Figure 4**).

### Gastrointestinal toxicity

#### Nausea

Any-grade nausea (5 RCTs; 5 treatments; 2787 patients) was significantly more common with niraparib (OR 5.13, 95% CI 3.49–7.53), olaparib (OR 5.33, 95% CI 1.64–17.35), senaparib (OR 3.49, 95% CI 1.85–6.55), and veliparib (OR 1.86, 95% CI 1.47–2.36) compared with placebo or chemotherapy. Indirect comparisons indicated a higher risk with niraparib than with veliparib (OR 2.75, 95% CI 1.75–4.33). For grade ≥3 nausea (4 RCTs; 4 treatments; 2474 patients), olaparib (OR 2.85, 95% CI 1.72–4.74) and veliparib (OR 3.70, 95% CI 2.19–6.26) were associated with significantly increased odds relative to placebo or chemotherapy(**Supplementary Figure 4**).

#### Vomiting

Any-grade vomiting (5 RCTs; 4 treatments; 2647 patients) showed higher odds with niraparib (OR, 3.58; 95% CI, 2.38–5.38), olaparib (OR, 3.89; 95% CI, 2.26–6.73), and veliparib (OR, 1.79; 95% CI, 1.45–2.21) versus placebo or chemotherapy (**Supplementary Figure 4**). Indirect comparisons showed higher odds with niraparib versus veliparib (OR, 2.00; 95% CI, 1.27–3.17) and olaparib versus veliparib (OR, 2.18; 95% CI, 1.21–3.91), suggesting relatively lower odds with veliparib. For grade ≥3 vomiting (5 RCTs; 4 treatments; 2647 patients), no pooled or indirect comparisons reached statistical significance.

#### Constipation

Any-grade constipation (5 RCTs; 4 treatments; 2647 patients) was associated with higher odds with niraparib (OR, 2.86; 95% CI, 1.98–4.13) compared with placebo or chemotherapy. Indirect comparisons showed higher odds with niraparib than with veliparib (OR, 2.78; 95% CI, 1.83–4.25), suggesting lower odds with veliparib (**Supplementary Figure 4**). For grade ≥3 constipation (4 RCTs; 3 treatments; 2257 patients), pooled comparisons showed higher odds with niraparib than with placebo or chemotherapy (OR, 11.87; 95% CI, 1.59–88.67). Indirect comparisons showed higher odds with niraparib versus veliparib (OR, 11.57; 95% CI, 1.52–88.37).

#### Diarrhoea

Any-grade diarrhoea (5 RCTs; 5 treatments; 2647 patients), pooled comparisons showed no statistically significant differences with niraparib (OR, 1.17; 95% CI, 0.81–1.70), olaparib (OR, 1.59; 95% CI, 0.99–2.56), veliparib (OR, 1.15; 95% CI, 0.93–1.42). For grade ≥3 diarrhoea (4 RCTs; 3 treatments; 2257 patients), no pooled or indirect comparisons reached statistical significance, including niraparib (OR, 0.16; 95% CI, 0.02–1.56) and veliparib (OR, 0.93; 95% CI, 0.46–1.85) versus placebo or chemotherapy (**Supplementary Figure 4**).

#### Abdominal pain

Any-grade abdominal pain (6 RCTs; 5 treatments; 2398 patients), pooled comparisons showed lower odds with niraparib compared with placebo or chemotherapy (OR, 0.63; 95% CI, 0.44–0.90). Indirect comparisons also showed lower odds with niraparib versus olaparib (OR, 0.46; 95% CI, 0.25–0.86) and veliparib (OR, 0.58; 95% CI, 0.37–0.92). For grade ≥3 abdominal pain (4 RCTs; 4 treatments; 1732 patients), no pooled or indirect comparisons reached statistical significance, including niraparib (OR, 0.65; 95% CI, 0.14–2.92), olaparib (OR, 2.02; 95% CI, 0.22–18.22), and veliparib (OR, 1.16; 95% CI, 0.63–2.15) versus placebo or chemotherapy (**Supplementary Figure 4**).

### Treatment-emergent adverse events leading to treatment discontinuation leading to any treatment discontinuation

Treatment-emergent adverse events leading to treatment discontinuation (3 RCTs; 2,155 patients), niraparib increased the odds of treatment discontinuation compared with placebo or chemotherapy (OR, 1.32; 95% CI, 1.08–1.62).

### TEAE leading to any treatment dose reduction

For treatment-emergent adverse events leading to dose reduction (3 RCTs; 2,155 patients), niraparib increased the odds of dose reduction compared with placebo or chemotherapy (OR, 2.44; 95% CI, 2.02–2.95).

### Narrative synthesis of additional safety outcomes

*Hardy-Bessard* et al. (2025) (36) reported no difference in overall survival (HR, 1.01; 95% CI, 0.86–1.19). Lower odds were observed for dostarlimab/placebo discontinuation (OR, 0.53; 95% CI, 0.39–0.72), treatment-related serious adverse events (OR, 0.67; 95% CI, 0.51–0.88), and treatment-related death (OR, 0.22; 95% CI, 0.05–0.98), whereas bevacizumab discontinuation was increased (OR, 1.57; 95% CI, 1.02–2.40). *González-Martín* et al. (2023) (41) reported higher odds of serious adverse events (OR, 2.40; 95% CI, 1.65–3.51), treatment-related serious adverse events (OR, 8.13; 95% CI, 3.92–16.88), and TEAEs leading to dose interruption (OR, 3.85; 95% CI, 2.76–5.35) with niraparib. *Mizuno* et al. (2023) (39) found no significant difference in serious adverse events (OR, 0.96; 95% CI, 0.72–1.27). *Vulsteke* et al. (2024) (48) reported higher odds of nausea without dose modification (OR, 2.37; 95% CI, 1.34–4.21). *Wu* et al. (2021) (44) reported higher odds of asthenia (OR, 22.37; 95% CI, 3.03–165.00).

*Mirza* et al. (2016) (42) reported higher odds of fatigue (OR, 1.44; 95% CI, 1.04–1.98) and grade 3–4 fatigue (OR, 14.63; 95% CI, 1.98–108.16), whereas grade 3–4 back pain was reduced (OR, 0.04; 95% CI, 0.01–0.19). *Valabrega* et al. (2024) (40) reported increased serious TEAEs, grade ≥3 TEAEs, and TEAEs leading to dose interruption in patients aged <65 years and ≥65 years receiving niraparib. *Moore* et al. (2018) (45) reported higher odds of fatigue or asthenia (OR, 1.53; 95% CI, 1.05–2.22), dose reduction (OR, 9.25; 95% CI, 3.31–25.86), and dose interruption (OR, 3.07; 95% CI, 1.87–5.05) with olaparib. *Wu* et al. (2024) (43) reported higher odds of fatigue with senaparib (OR, 5.64; 95% CI, 2.92–10.86). **Supplementary Table 4** represents the details of studies included in narrative synthesis.

### Treatment rankings

Overall, P-score ranking identified olaparib as the highest-ranked treatment for progression-free survival in younger and older patients, whereas senaparib ranked highest among adults aged ≥18 years. Placebo or chemotherapy demonstrated the most favourable ranking across most safety outcomes, including neutropenia, thrombocytopenia, nausea, vomiting, constipation, diarrhoea, TEAEs, treatment discontinuation, and dose reduction. Veliparib demonstrated favourable rankings for several hematologic and gastrointestinal toxicities. In contrast, niraparib showed less favourable rankings for most hematologic and treatment-related outcomes, but ranked highest for abdominal pain and grade ≥3 diarrhoea. Olaparib ranked highest for grade ≥3 thrombocytopenia. No significant differences were observed for grade ≥3 vomiting. Placebo or chemotherapy ranked highest for treatment discontinuation and dose reduction.

### Influence analysis

We performed influence analysis for outcomes with more than five studies. Excluding Coleman RL_2019 reduced the evidence for the Placebo/Chemo-only versus Veliparib comparison to one study in the younger population and in patients aged ≥18 years, while the older population network lost the Veliparib node. Excluding X. H. Wu et al. (2021) (44) did not change the network structure for any-grade vomiting, diarrhoea, constipation, grade ≥3 anaemia, or any-grade TEAEs. Removing Wu et al. (2024) (43) changed the network structure for any-grade and grade ≥3 neutropenia, any-grade thrombocytopenia, and any-grade nausea by removing the senaparib comparison. Excluding Mirza et al. (2016) (42) removed the niraparib comparison in grade ≥3 thrombocytopenia (**Supplementary Figure 5**).

## Discussion

This network meta-analysis included 13 randomized controlled trials evaluating PARP inhibitors (PARPi) in advanced ovarian cancer, with emphasis on age-stratified efficacy and safety outcomes. Overall, PARPi significantly improved progression-free survival (PFS) across all evaluated age groups. In younger patients, olaparib, rucaparib, and niraparib significantly reduced the risk of disease progression, while similar benefits were observed in older patients aged ≥65 years. Among adults aged ≥18 years, senaparib, veliparib, and olaparib also demonstrated significant PFS benefit. Olaparib achieved the highest P-score ranking in younger and older patients, whereas senaparib ranked highest in the overall adult population. However, P-score rankings reflect probabilistic hierarchy estimates and should not be interpreted as definitive evidence of clinical superiority. Substantial heterogeneity across studies and the absence of formal treatment-by-age interaction analyses further warrant cautious interpretation.

Safety analyses showed increased hematologic and gastrointestinal toxicities with several PARPi regimens. Niraparib and olaparib were associated with higher rates of grade ≥3 anaemia, while niraparib, senaparib, olaparib, and veliparib showed increased risks of neutropenia and thrombocytopenia. Nausea, vomiting, and constipation were also more frequent with PARPi, particularly niraparib and olaparib. Placebo or chemotherapy demonstrated the most favourable safety rankings across most outcomes.

Findings from prior systematic reviews and network meta-analyses are largely consistent with the results of the present study. Cai et al. (2021) (49), Xiao et al. (2025) (50), and Huang et al. (2024) (51) reported that PARP inhibitors provide meaningful improvements in progression-free survival across multiple cancer types, while demonstrating substantial variability in safety profiles among agents. These observations align with the current network meta-analysis, which showed consistent efficacy benefits across age groups in advanced ovarian cancer, accompanied by differences in hematologic toxicity. Several studies have highlighted higher risks of anaemia and other hematologic adverse events with specific PARP inhibitors. Cai et al. (2021) (49), Liu et al. (2022) (52), and Xiao et al. (2025) (51) reported increased rates of severe anaemia with niraparib and olaparib, whereas veliparib showed comparatively lower hematologic toxicity. These findings parallel the present results, in which olaparib and niraparib were associated with higher odds of grade 3 or higher anaemia, while veliparib demonstrated smaller effect estimates.

Evidence from prostate and endometrial cancer further supports these patterns. Huang et al. (2024) (51) and Li et al. (2026) (53) reported improved disease control with PARP inhibitors in prostate cancer, particularly with olaparib-based regimens, but noted increased treatment-related toxic effects. Similarly, Villacampa et al. (2023) (54) observed improved efficacy with PARP inhibitor–based strategies in endometrial cancer, accompanied by higher rates of adverse events.

Recent real-world evidence further supports the feasibility of PARPi therapy in older patients. Apostol et al. (2025) (55) reported comparable progression-free survival across age groups, including patients aged ≥75 years, despite less favourable baseline clinical characteristics. Similarly, Kim et al. (2025) (56) demonstrated that proactive dose modification strategies in older patients with HRD-positive advanced ovarian cancer improved tolerability without compromising progression-free survival. Masvidal Hernández et al. (2024) (57) also highlighted the clinical benefit of PARPi in older adults while emphasising the limited availability of quality-of-life data.

A major strength of this analysis is its age-stratified approach, which addresses an important evidence gap because older adults remain underrepresented in randomised trials of advanced ovarian cancer. Despite concerns regarding altered pharmacokinetics, comorbidities, and polypharmacy, PFS benefits were also observed in patients aged ≥65 years. These findings support treatment decisions based on overall fitness and clinical characteristics rather than chronological age alone.

No direct head-to-head randomised trials comparing PARPi were identified; therefore, comparative estimates were derived primarily from indirect evidence. Substantial heterogeneity and overlapping confidence intervals were observed across several comparisons. The observed heterogeneity likely reflects differences in disease setting, treatment strategy, prior therapy exposure, BRCA/HRD status, concomitant bevacizumab use, and follow-up duration across studies.

Several limitations should be considered when interpreting these findings. Substantial heterogeneity was observed across several efficacy and safety networks, particularly for progression-free survival outcomes. In addition, network meta-analysis relies on the assumptions of homogeneity, consistency, and transitivity. Formal inconsistency assessment was limited because most treatment networks exhibited a predominantly star-shaped geometry without closed loops. The transitivity assumption may have been challenged by differences in patient populations, biomarker status, treatment strategies, and comparator regimens across studies. Although grouping placebo- and chemotherapy-containing control regimens was necessary to maintain network connectivity, these interventions are not clinically equivalent and may have introduced uncertainty into indirect treatment estimates. Several safety estimates also demonstrated wide confidence intervals, likely reflecting sparse-event data and limited precision.

The evidence network relied largely on indirect comparisons because no direct head-to-head randomized trials of PARPi were available. Furthermore, age subgroup definitions varied across studies, potentially introducing ecological bias. Most included trials enrolled relatively fit patients and provided limited information regarding frailty, functional status, polypharmacy, comorbidity burden, and geriatric assessment measures, which may restrict the applicability of these findings to the broader older population. Clinically important outcomes, including overall survival, quality of life, dose modifications, and treatment discontinuation, were inconsistently reported. Publication bias could not be formally assessed because fewer than 10 studies were available within most comparison networks, making funnel plots unreliable. Consequently, small-study effects cannot be excluded. In addition, the certainty of evidence was not formally evaluated using approaches such as CINeMA or GRADE, which may limit confidence in the comparative treatment estimates.

Future studies should prioritise enrolment of elderly and frail patients and incorporate comprehensive geriatric assessment tools, patient-reported outcomes, and real-world tolerability data. Ongoing geriatric-focused studies, including FRAGINOC and PARIB-OLD-PRO² (58), may provide further insight into frailty assessment, treatment tolerability, and optimization of PARPi therapy in older adults with advanced ovarian cancer.

## Conclusion

We found PARP inhibitors improved progression-free survival across age groups, including patients aged ≥65 years. Progression-free survival benefits were observed in both younger and older patients. However, PARP inhibitors were associated with increased hematologic and gastrointestinal toxicities. These findings support consideration of PARP inhibitors in appropriately selected older patients. Further studies should assess geriatric outcomes, long-term safety, and patient-reported outcomes.

## Data Availability

The original contributions presented in the study are included in the article/**Supplementary Material**, further inquiries can be directed to the corresponding author/s.
